# Automation of PacBio SMRTbell NGS library preparation for bacterial genome sequencing

**DOI:** 10.1186/s40793-017-0239-1

**Published:** 2017-03-23

**Authors:** Nguyet Kong, Whitney Ng, Kao Thao, Regina Agulto, Allison Weis, Kristi Spittle Kim, Jonas Korlach, Luke Hickey, Lenore Kelly, Stephen Lappin, Bart C. Weimer

**Affiliations:** 1 Population Health and Reproduction Department, School of Veterinary Medicine, University of California-Davis, Davis, CA USA; 2 Genentech, S. San Francisco, CA USA; 3 University of California-San Francisco, San Francisco, CA USA; 4 Pacific Biosciences, Menlo Park, CA USA; 5 Agilent Technologies, Inc., Santa Clara, CA USA

**Keywords:** PacBio SMRTbell NGS library preparation, Bacterial genomic DNA, Automation, NGS workstation, TapeStation System, Bioanalyzer

## Abstract

**Background:**

The PacBio RS II provides for single molecule, real-time DNA technology to sequence genomes and detect DNA modifications. The starting point for high-quality sequence production is high molecular weight genomic DNA. To automate the library preparation process, there must be high-throughput methods in place to assess the genomic DNA, to ensure the size and amounts of the sheared DNA fragments and final library.

**Findings:**

The library construction automation was accomplished using the Agilent NGS workstation with Bravo accessories for heating, shaking, cooling, and magnetic bead manipulations for template purification.

The quality control methods from gDNA input to final library using the Agilent Bioanalyzer System and Agilent TapeStation System were evaluated.

**Conclusions:**

Automated protocols of PacBio 10 kb library preparation produced libraries with similar technical performance to those generated manually. The TapeStation System proved to be a reliable method that could be used in a 96-well plate format to QC the DNA equivalent to the standard Bioanalyzer System results. The DNA Integrity Number that is calculated in the TapeStation System software upon analysis of genomic DNA is quite helpful to assure that the starting genomic DNA is not degraded. In this respect, the gDNA assay on the TapeStation System is preferable to the DNA 12000 assay on the Bioanalyzer System, which cannot run genomic DNA, nor can the Bioanalyzer work directly from the 96-well plates.

## Introduction

Increased throughput from the use of next generation sequencing methods has revealed new information about the function and structure of bacterial genomes. The use of short reads to produce draft genomes leads to problems with GC content bias and repeat regions that make it tedious to produce closed genome assemblies. This technical note discusses the PacBio RS II approach using a single molecule, real-time DNA sequencing approach to improve genome assembly through extra-long read lengths. By reducing the number of contigs, the accuracy of the *de novo* assembly of bacterial whole genomes is facilitated. The real-time technology of the PacBio RS II allows determination of not only the full, closed, gDNA sequence, but also epigenetic modifications and plasmid DNA sequence simultaneously.

The 100K Pathogen Genome Project [1] is using the PacBio 10 kb SMRTbell Template Preparation kit to produce 1,000 closed genomes. The scale of this project required automation of the construction of the sequencing (SMRTbell™) library. To prepare libraries for sequencing in this way, gDNA must be cut into fragments to a target size of 10 kb. Critical to generating long sub-reads, it is important to start with high quality gDNA input in order to shear the gDNA into the target fragment size to ensure the correct concentrations during library construction to react properly with the concentrations of reagents in each of the given steps. Gel electrophoresis is a low-resolution traditional method with sizing against a ladder and determining concentration on an agarose gel by comparing peak density to a standard, and since it cannot be automated, is not suitable for a project of this size. Another way to measure size and concentration is to use the Agilent 2100 Bioanalyzer with the DNA 12000 assay, but the instrument only runs 12 samples at a time and cannot be automated. We will discuss the automation of preparation of libraries with the SMRTbell Template Preparation kit as well as analysis of gDNA, fragmented DNA and the final libraries ready for sequencing with both the Agilent electrophoresis platform: Agilent 2100 Bioanalyzer System using the DNA 12000 assay and the Agilent TapeStation System using the genomic DNA ScreenTape and matching reagents.

## Procedure

*Campylobacter jejuni* *,* *Listeria monocytogenes* *,* *Vibrio fluvialis* and *Salmonella enterica* serovar. *Enteritidis* were cultured in appropriate culture medium and growing condition listed in Tables 1 and 2. Bacteria were cultured on the appropriate agar and pellets were made for extraction. DNA was extracted from the cell pellets using a kit and clean-up was accomplished with a spin column [2–4]. Absorbance ratios at 260/280 and 260/230 were measured with a NanoDrop 2000 UV-vis spectrophotometer (Thermo Fisher Scientific, Waltham MA). A Qubit 2.0 Fluorometer (Q32866) was used with a Qubit dsDNA HS Assay Kit (Q32854, both from Invitrogen, Carlsbad CA) to measure the gDNA concentration and confirm DNA input of 10 μg before shearing. The initial evaluation of the quantity and size distribution of the purified gDNA was with the Agilent 2200 TapeStation Nucleic Acid System (G2965AA) controlled by Agilent 2200 TapeStation Software A.01.05, using the Agilent Genomic DNA ScreenTape (5067–5365) and the Agilent Genomic DNA Reagents (5067–5366) with samples drawn from a 96-well plate [5, 6]

**Table 1:**
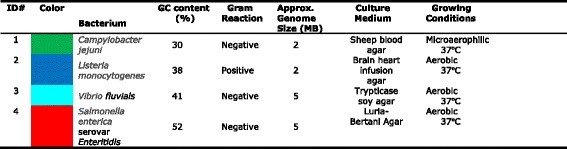
Organisms used in this study

**Table 2:**
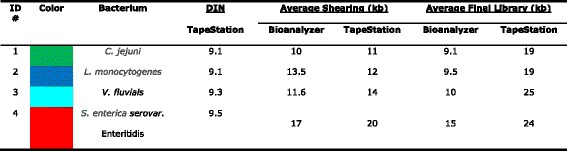
gDNA quality, average shearing size and average final library for each bacterium

Genomic DNA was sheared using the Covaris g-TUBE device (520079) according to the manufacturer specifications [7]. After fragmentation, DNA was evaluated with the TapeStation System with the Genomic DNA assay and also with the Agilent 2100 Bioanalyzer System with the Agilent DNA 12000 assay (5067–1508) [8, 9]. Both of these methods have minimal sample consumption and return both sizing and quantitation. The sheared gDNA sample input was normalized for all samples between 1–5 μg into library construction for PacBio SMRTbell 10 kb Library Preparation.

**Fig. 1:**
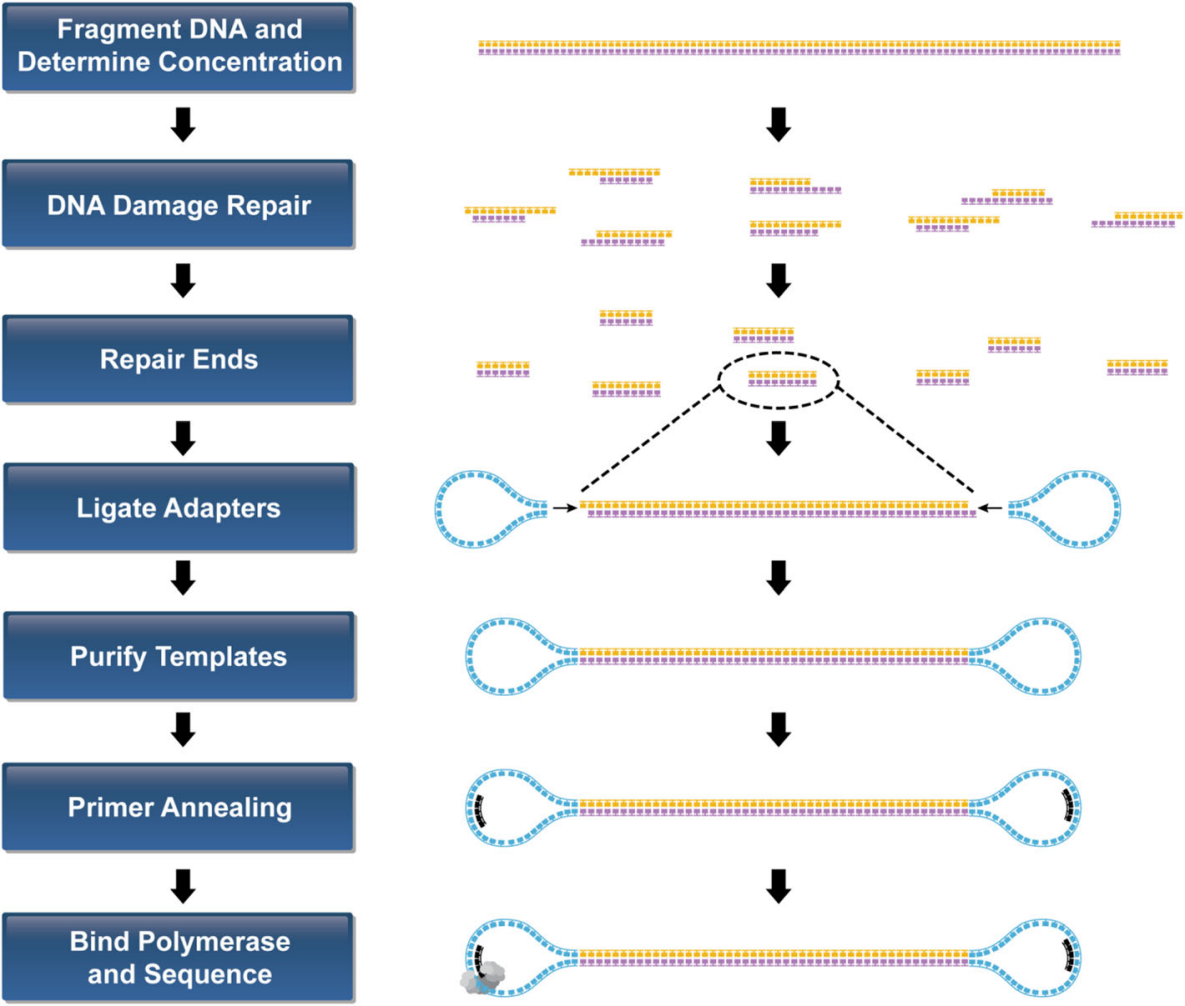
PacBio SMRTbell Template Preparation Workflow for PacBio RS II system. PacBio SMRTbell Template Preparation Workflow for PacBio RS II system. This workflow is used to prepare libraries from fragmented and concentrated DNA using Covaris g-TUBE and concentrated using the AMPure magnetic beads before following PacBio SMRTbell 10 kb Library Preparation procedures

The SMRTbell Template Preparation kit from Pacific Biosciences (Menlo Park CA) was used on the Agilent NGS Workstation (G5522A, Agilent Technologies, Santa Clara CA). The workflow to construct the final DNA libraries for sequencing is shown in Fig. 1 and involved automation of these steps:

1. Determination of the quality of the gDNA
2. Fragment gDNA using a Covaris g-TUBE device
3. QC the sizing and adjust the concentration
4. Repair DNA damage and repair ends of fragmented DNA
5. Purify the DNA
6. Blunt-end ligate using blunt adapters
7. Purify template for submission to a sequencer

**Fig. 2:**
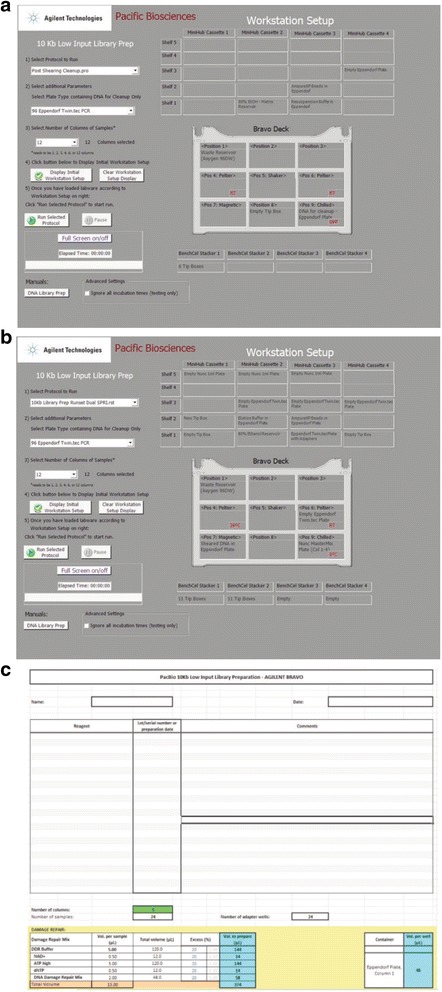
VWorks protocols and Excel workbook for PacBio Library Preparation. VWorks protocols and Excel workbook for PacBio Library Preparation method provide an interactive, visual layout for the end user. **a** Post Shearing Cleanup Form. **b** 10 kb Library Prep Runset Dual SPRI Form. **c** PacBio Library Excel Workbook

In Fig. 2, A (Post Shearing Clean-up) and B (10kb Library Prep Runset Dual SPRI) are two of the VWorks protocol graphical user interfaces that help with the NGS Workstation setup and deck layout to optimize the use of reagent volumes. This interface allows the user to view the progress of the procedure. In Fig. 2c, the Excel template assists with laying out the reagent amounts and calculations, and provides a record of each batch of reagents preparation and lot numbers.

With the automation, this workflow takes about 7 h for post-shearing clean-up and library construction. Once the PacBio 10 kb library is made, the final library was confirmed with the Agilent 2200 TapeStation with the Genomic DNA ScreenTape assay and the Agilent 2100 Bioanalyzer System with the Agilent DNA 12000 assay to determine the size of the library. Libraries are quantified using a Qubit 2.0 Fluorometer (Q32866) with a Qubit dsDNA HS Assay Kit (Q32854, both from Invitrogen, Carlsbad CA) to measure the library concentration before submission to the sequencing facility. The sequencing facility anneals sequencing primer and binds polymerase to the SMRTbell templates before loading the library onto the PacBio RS II.

## Discussion

The genomic DNA isolated from four model organisms with a range of GC content were made into libraries prepared on the Agilent NGS Workstation with PacBio SMRTbell Template Preparation kit for sequencing on the PacBio RSII. Finished sequences showed GC content very close to the known GC content, thus showing this process produced minimal bias (Table 1).

**Fig. 3:**
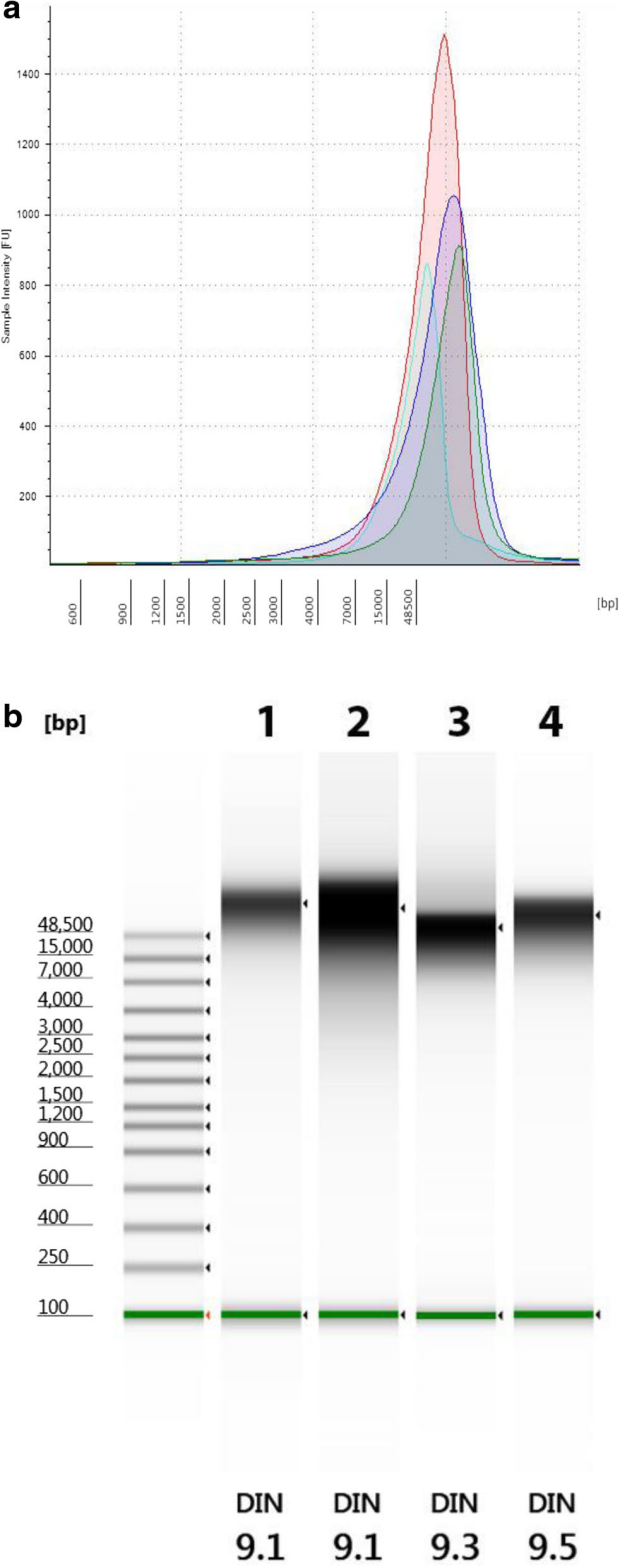
Quantitation of Genomic DNA. Electropherogram (**a**) and gel image (**b**) of high molecular weight gDNA from Agilent 2200 TapeStation using the Genomic DNA ScreenTape System. Campylobacter (*green*), *Listeria* (*blue*), *Vibrio* (*aqua*), and *Salmonella* (*red*). *Green lines* at the *bottom* of the gel image are internal standards added to permit quantitation. *Lower* marker is not shown in the electropherogram

For the best results to produce genomic sequences, it is important the starting material be relatively free of organics and protein, and be at least 50 kilobases to insure long fragments can be obtained for sequencing. The microbes used are listed in Table 1 and include four genera of varying length and GC content. The organisms were cultured and genomic DNA was extracted followed by spin column clean-up. The quality of the gDNA was measured with the NanoDrop and the 260/280 nm and the 260/230 nm ratios were calculated. The 260/280 nm ratio and 260/230 nm ratio of 1.8 was the requirement for further use of each extraction. The Agilent 2200 TapeStation System with the Genomic DNA assay was used to assess size and concentration of each sample as shown in Fig. 3, where an electropherogram overlay and virtual gel images are shown for the four model organisms, together with the DIN calculated by the TapeStation software. The DNA Integrity Number (DIN) helped establish a cut-off for the suitability of the gDNA for further work and can be useful for library construction.

**Fig. 4:**
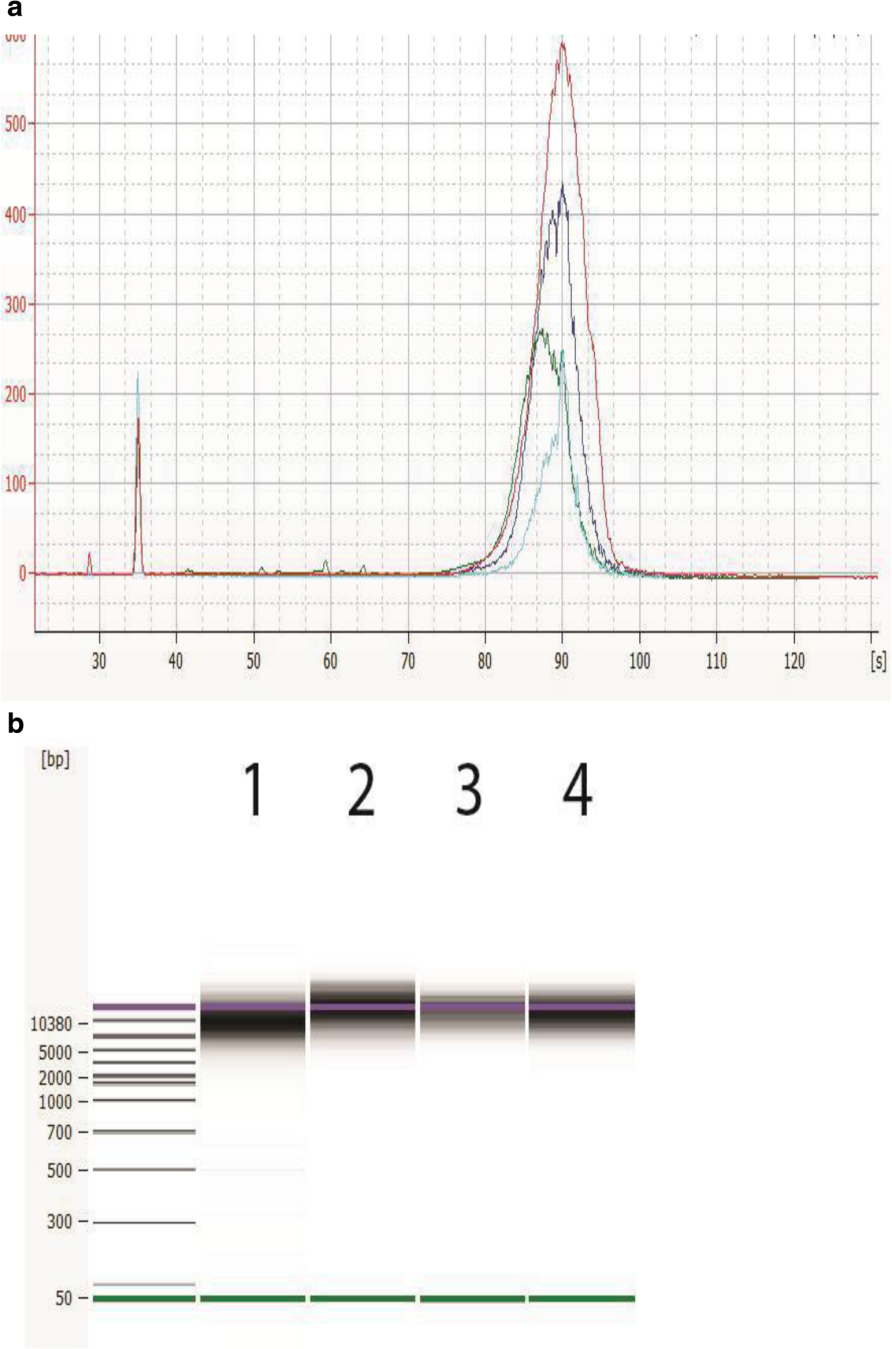
Appearance of sheared DNA from Agilent 2100 Bioanalyzer analysis. Representative electropherogram (**a**) and virtual gel (**b**) are used for visual inspection (generated with the Agilent 2100 Bioanalyzer system with the DNA 12000 Kit) of sheared bacterial genomic DNA with average shearing size for *Campylobacter* (*green*, 10 kb), *Listeria* (*blue*, 13.5 kb), *Vibrio* (*aqua*,11.6 kb), and *Salmonella* (*red*, 17 kb). Peaks near 35 are the *lower* marker internal standard for the DNA 12000 kit. A typical electropherogram using the Agilent Bioanalyzer 2100 DNA 12000 kit shows the *lower* marker at 35 s and the *upper* marker at 90 s. The sheared DNA and the *red* *upper* marker, seen in the gel image, co-elute together

**Fig. 5:**
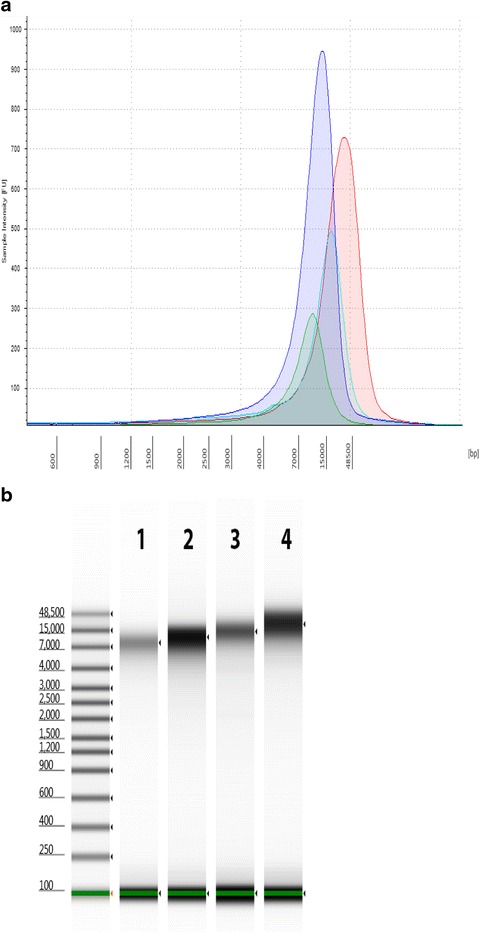
Appearance of sheared DNA from Agilent 2200 TapeStation analysis. Representative electropherogram (**a**) and virtual gel (**b**) of sheared bacterial genomic DNA was generated with the Agilent 2200 TapeStation genomic DNA Kit with the average shearing size for *Campylobacter* (*green*, 16 kb), *Listeria* (*blue*, 12 kb), *Vibrio* (*aqua*, 14 kb), and *Salmonella* (*red*, 20 kb). *Green lines* at the bottom of the gel image are internal standards added to permit quantitation. *Lower* marker is not shown in the electropherogram

Following qualification of the gDNA, the next step is to shear the gDNA into the target fragment size required for library construction using a Covaris g-TUBE device according to manufacturer instructions. It is important to check the fragment size and the DNA amount prior to proceeding with the library construction. Traditionally, this has been done with the Agilent 2100 Bioanalyzer system with the DNA 12000 kit and these results are shown in Fig. 4 as overlaid electropherograms and a virtual gel image together with the sizing ladder provided. The DNA 12000 kit uses both a lower and an upper marker as internal standard. For these samples with a target size of 10 kb, the DNA fragments usually run together with the upper marker, which can be easily seen on the gel image since it is shown in red. In the electropherogram view, the upper marker is the sharp peak at 90 s. The Agilent 2200 TapeStation System with the gDNA ScreenTape assay can qualify the fragment size too, and this is shown in Fig. 5. The assay has a larger range to quantify genomic DNA larger than 12 kb with no upper marker and can run directly out of a 96 well plate. It is important to determine the correct sizing, in order for the sequencing facility to properly load the libraries on the sequencer.

**Fig. 6:**
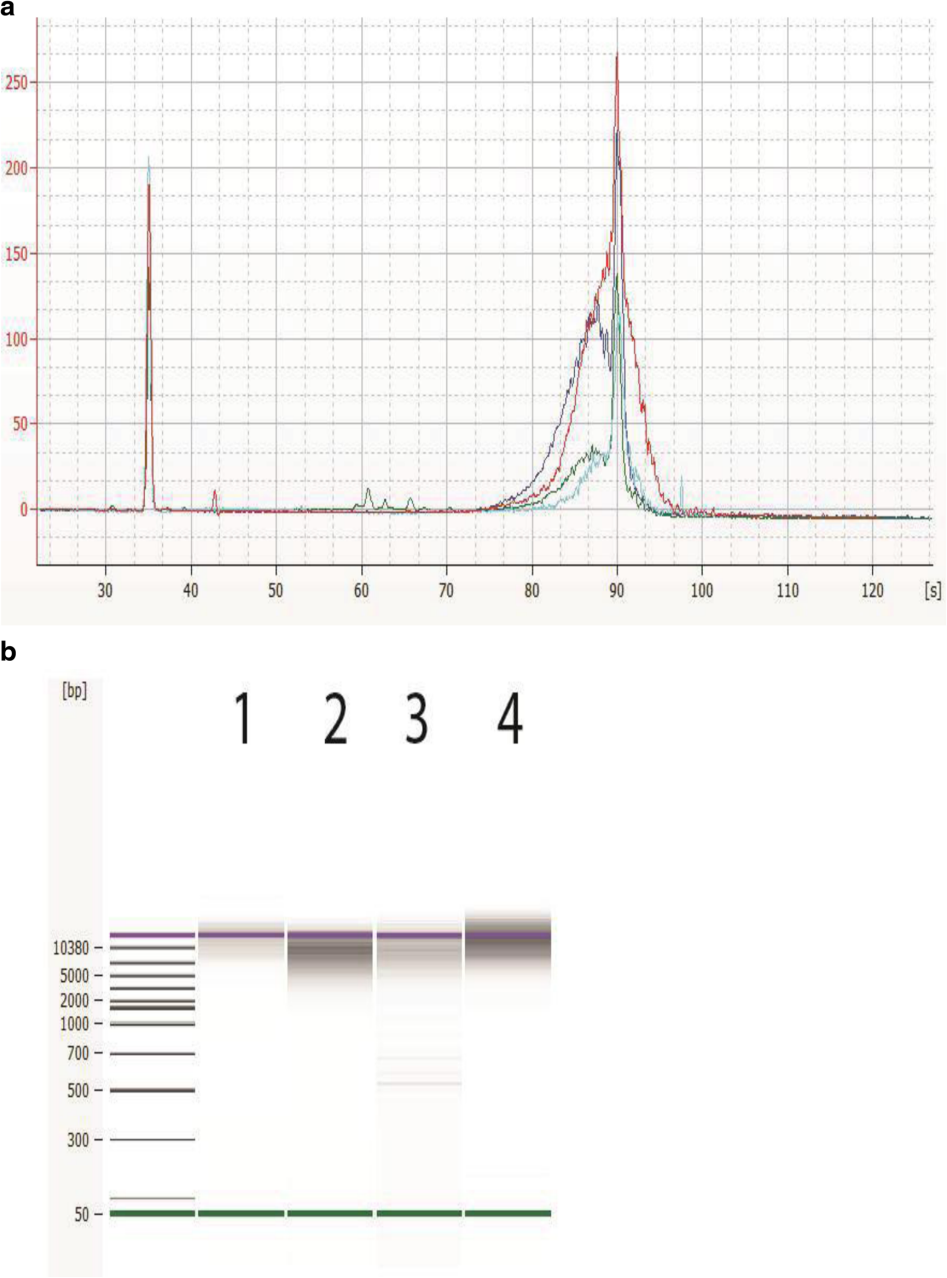
Appearance of DNA libraries from Agilent 2100 Bioanalyzer analysis. Representative electropherogram (**a**) and virtual gel (**b**) used for visual inspection (generated with the Agilent 2100 Bioanalyzer system with the DNA 12000 Kit) of DNA libraries sizes prepared for sequencing with the PacBio SMRTbell 10 kb Template Preparation Kit on the Agilent NGS Workstation. A typical electropherogram using the Agilent bioanalyzer 2100 DNA 12000 kit shows the *lower* marker at 35 s and the *upper* marker at 90 s. The DNA libraries and the *upper* marker co-elutes with each other, the sharper peak is the *upper* marker, shown in *red* on the gel image. The average library sizes are: *Campylobacter* (*green*, 9.1 kb), *Listeria* (*blue*, 9.5 kb), *Vibrio* (*aqua*, 10 kb), and *Salmonella* (*red*, 15 kb)

**Fig. 7:**
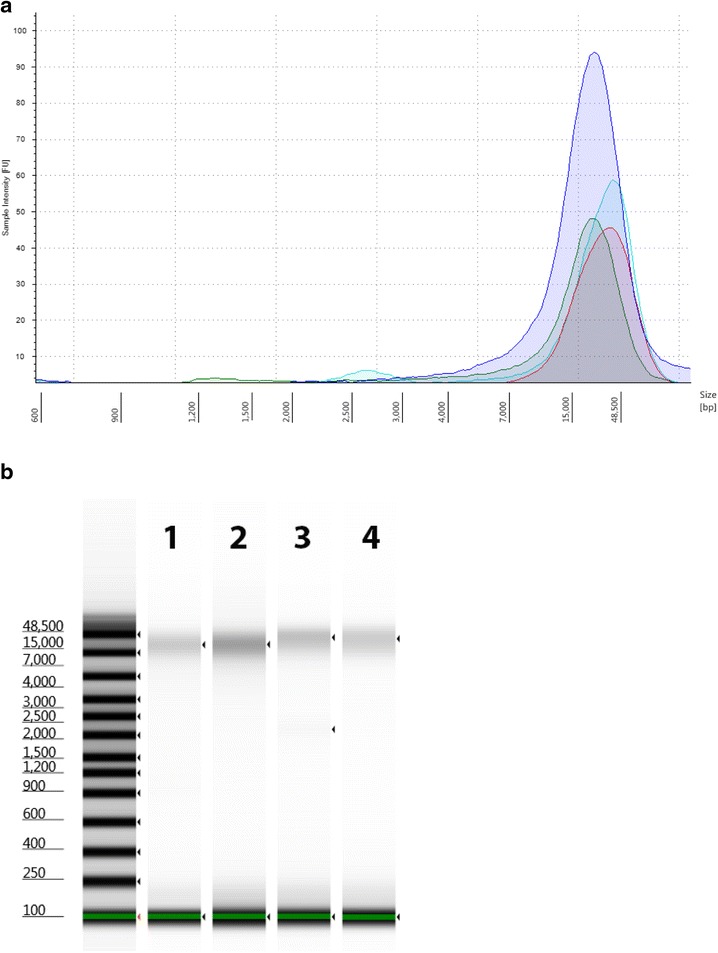
Appearance of DNA libraries from Agilent 2200 TapeStation analysis. Representative electropherogram (**a**) and virtual gel (**b**) of DNA libraries sizes (generated with the Agilent 2200 TapeStation DNA genomics Kit) prepared for sequencing with the PacBio SMRTbell 10kb Template Preparation Kit on the Agilent NGS Workstation. The average library size for *Campylobacter* (*green*, 16 kb), *Listeria* (*blue*, 12 kb), *Vibrio* (*aqua*, 14 kb), and *Salmonella* (*red*, 20 kb) is displayed on the software screen. *Green lines* at the *bottom* of the gel image are internal standards added to permit quantitation. *Lower* marker is not shown in the electropherogram

Libraries are made following the PacBio SMRTbell 10kb Library Preparation on the Agilent NGS Workstation and traditionally confirmed with the Agilent 2100 Bioanalyzer System with the DNA 12000 kit, shown in Fig. 6. Thus, with SMRTbell templates around 10 kb in size, it’s difficult to determine the correct sizing for those libraries as these constructs also run with the upper marker shown in red on the virtual gel images. Since the Agilent 2200 TapeStation System can size larger fragments up to 60 kb, it can determine the size more accurately, as shown in Fig. 7.

## Conclusion

The PacBio SMRTbell 10 kb Library preparation kit can be used with automation such as the Agilent Bravo to prepare microbial libraries with minimal GC bias. QC of the starting DNA and the required fragment preparation with the Covaris g-TUBE can be done with the Agilent 2200 TapeStation and the gDNA ScreenTape assay directly from the 96 well plates used by the Bravo to prepare the libraries.

## Abbreviations

DIN: DNA integrity number
gDNA: Genomic DNA
NGS: Next generation sequencing
SMRT: Single molecule, real-time
